# SIRT3 inhibitor 3-TYP exacerbates thioacetamide-induced hepatic injury in mice

**DOI:** 10.3389/fphys.2022.915193

**Published:** 2022-07-18

**Authors:** Chunxia Shi, Fangzhou Jiao, Yao Wang, Qian Chen, Luwen Wang, Zuojiong Gong

**Affiliations:** ^1^ Department of Infectious Diseases, Renmin Hospital of Wuhan University, Wuhan, China; ^2^ Department of Infectious Diseases, Zhongnan Hospital of Wuhan University, Wuhan, China

**Keywords:** 3-TYP, SIRT3, thioacetamide, acute liver failure, inflammation

## Abstract

The purpose of the study was to explore the effects of SIRT3 inhibitor 3-TYP on acute liver failure (ALF) in mice and its underlying mechanism. The mice were treated with thioacetamide (TAA, 300 mg/kg) for inducing ALF model. 3-TYP (50 mg/kg) was administered 2 h prior to TAA. The liver histological changes were measured by HE staining. Blood samples were collected for analysis of alanine aminotransferase (ALT) and aspartate aminotransferase (AST). MDA and GSH were used to evaluate the oxidative stress of liver. The expression levels of inflammatory cytokines (TNF-α and IL-1β) were measured by ELISA and Western blotting. The cell type expression of IL-1β in liver tissue was detected by immunofluorescent staining. The expression of SIRT3, MnSOD, ALDH2, MAPK, NF-κB, Nrf2/HO-1, p-elF2α/CHOP, and cleaved caspase 3 was determined by Western blotting. TUNEL staining was performed to detect the apoptosis cells of liver tissues. 3-TYP exacerbated the liver injury of ALF mice. 3-TYP increased the inflammatory responses and activation of MAPK and NF-κB pathways. In addition, 3-TYP administration enhanced the damage of oxidative stress, endoplasmic reticulum stress, and promoted hepatocyte apoptosis in ALF mice. 3-TYP exacerbates thioacetamide-induced hepatic injury in mice. Activation of SIRT3 could be a promising target for the treatment of ALF.

## Introduction

Histone deacetylases are key modulators by posttranslational deacetylation modifications, which are involved in various physiologic functions. Sirtuins are special enzyme of histone deacetylases because their catalytic mechanism needs nicotinamide adenine dinucleotide (NAD+). The human sirtuins are divided into four classes. Class I is comprised of SIRT1, SIRT2, and SIRT3, which close homology to yeast Sir2. SIRT4 belongs to class II Sirtuin, and SIRT5 is class III Sirtuin. SIRT6 and SIRT7 are class IV (Zhang et al., 2020). Among them, SIRT3 is a special member mainly located in mitochondria (Salvatori et al., 2017). As a mitochondrial sirtuin, SIRT3 could modify the enzymatic activities of many mitochondrial proteins by acetylation modification (Nogueiras et al., 2012). In addition, increasing studies have reported that SIRT3 could regulate mitochondria biological function by directly interaction with 84 mitochondria proteins (Zhang et al., 2020). Apart from the regulation of mitochondria function, an increasing number of studies have reported that SIRT3 could regulate numerous functions, including aging, DNA repair, metabolism, tumorigenesis, oxidative stress, and inflammation (Ansari et al., 2017).

Acute liver failure (ALF) is a high mortality liver disease characterized by massive hepatocyte necrosis. It is caused by various factors, such as hepatotoxic drugs, hepatitis virus, and metabolic disorders. So far, liver transplantation is still the lifesaving treatment option. It is imperative to find a new solution for ALF, due to the costly expenses of surgery and scarcity of liver donors. The pathological feature of liver tissues is massive liver cell necrosis accompanied with inflammatory cells. The tissue inflammation plays a central role in the pathogenesis of liver failure (Triantafyllou et al., 2018). Mitogen-activated protein kinase (MAPK) pathways and nuclear factor kappa *β* (NF-κβ) are important signaling pathways, which mediate the inflammatory process in several inflammation diseases (DiDonato et al., 2012; Kim and Choi, 2015). MAPK pathways are comprised of P38, JNK, and ERK. Activation of MAPK pathways by upregulation of the phosphorylation levels of three members. The activation of MAPK pathways and NF-κβ mediate the genes transcription of proinflammatory factors, which cause the inflammation process (Dong et al., 2002).

Oxidative stress is an imbalance of oxidation reduction system in tissue and cell and characterized by accumulation of reactive oxygen species (ROS). Oxidative stress involved in various acute and chronic liver diseases, including acute liver failure (Bruck et al., 2004), non-alcoholic fatty liver diseases (NAFLD), alcoholic liver diseases (ALD) (Li et al., 2015), hepatic encephalopathy, and hepatic fibrosis (Cichoz-Lach and Michalak, 2014). In addition, one study found that an antioxidant (N-acetylcysteine amide) ameliorated the damage of liver function, histopathology, and oxidative stress in acetaminophen -induced hepatotoxicity (Khayyat et al., 2016). Endoplasmic reticulum stress (ERS) is the pathologic process of accumulation of misfolded or unfolded proteins. Increasing evidence support that the contribution of ERS to various liver diseases, including NAFLD, ALD, ischemia reperfusion injury (Dara et al., 2011), acute liver injury (Yan M. et al., 2018).

A previous study has demonstrated that SIRT3 knockout (KO) mice reduced the acetaminophen-induced hepatotoxicity (Lu et al., 2011). However, another study has reported that SIRT3 KO mice exacerbated the carbon tetrachloride (CCl_4_)-induced acute liver injury model (Li et al., 2019). The opposite results are arousing our research interest in SIRT3 and ALF. In addition to acetaminophen and CCl_4_, thioacetamide (TAA) was also common hepatotoxic drug (Tunon et al., 2009; Jaeschke et al., 2013). In our previous study, TAA could be established a better model of liver failure (Jiao et al., 2019). Thus, TAA was used to induce the acute liver injury model in present study. Previous studies have reported that SIRT3 inhibitor 3-TYP could effectively downregulate SIRT3 *in vivo* study (Ye et al., 2019). In this research, we investigated the effect and potential mechanism of selective SIRT3 inhibitor (3-TYP) in TAA-induced mice liver injury.

## Materials and methods

### Drugs and chemicals

3-TYP (S8628) was obtained from MedChemExpress (China). TAA 163678) were obtained from Sigma-Aldrich (United States). Phospho-eIF2α (#3398), eIF2α (#5324), CHOP(#2895), phospho-JNK (#4668), JNK (#9252), phospho-ERK1/2 (#4370), ERK1/2 (#9102), phospho-P38 (#4511), P38 (#8690), phospho-NF-κB-p65 (#3033), NF-κB-p65 (#8242), Nrf2 (#12721), Lamin B1 (#13435), HO-1 (#43966), Cleaved caspase 3 (#9664), TNF-α (#11948), IL-1β (#12426), SIRT1 (#8469), SIRT2 (#12672), SIRT3 (#5490), MnSOD (#13194), and GAPDH (# 5174) were obtained from Cell Signaling Technology (CST) (United States), ALDH2 (15310-1-AP) antibody was obtained from Proteintech (China).

### Animals study

C57BL/6 J mice (7–8 weeks; 20–22 g) were obtained from Beijing Vital River Laboratory Animal Technology (Beijing, China). All mice use and care protocols were authorized by Remmin Hospital of Wuhan University ethics committee (WDRM (F) 20181018). Mice were randomly divided into three groups (*n* = 10 each): normal, TAA, and 3-TYP group. As described in the previous study, TAA was used to establish the mice ALF model (Jiao et al., 2019). The TAA group were administered with 300 mg/kg TAA each mouse. 3-TYP was administrated by intraperitoneal injection in 3-TYP group before TAA injection 2 h. The dosage of 3-TYP (50 mg/kg) was selected according to previous published studies (Zhai et al., 2017; Ye et al., 2019). The survival time of all mice was observed during the process of experiment. At 24 h time point after TAA, all mice were anesthetized and sacrificed, and the samples of liver, serum were harvested.

### Histological examination

The histological changes of liver tissues were performed by hematoxylin and eosin (HE), according to standard staining methods (Feldman and Wolfe, 2014). Briefly, the tissues were fixed in 10% formalin, embedded in paraffin, and sectioned at 5 µm. The section was stained with HE. The results were observed under light microscope (Olympus, Japan).

### Blood measurements

Mice blood were collected for detection the levels of alanine aminotransferase (ALT), aspartate aminotransferase (AST), TNF-α, and IL-1β. The levels of ALT and AST were detected by Hitachi Automatic Analyzer (Japan). The serum levels of TNF-α (JL10484) and IL-1β (JL18442) were detected by ELISA kits (Jianglai biotech, Shanghai).

### Assessment of liver oxidative stress markers

The levels of malondialdehyde (MDA) and glutathione (GSH) were used to measure the degree of liver oxidative stress. Lipid peroxidation product level of lipid peroxides (MDA, S0131S, Beyotime, China) were estimated by using TBARS assay. The reduced glutathione (GSH, S0053, Beyotime, China) level was measured by according to the method of study (Beutler et al., 1963).

### Immunofluorescence staining

Immunofluorescence staining was used to detect the protein expression of CD68 and IL-1β in liver tissues. Briefly, the slices of liver tissue were blocked with goat serum for 1 h. Then, the liver slices were incubated with anti-CD68 antibody (1:100 dilutions, ab201340, Abcam, United States) for 24 h. Then, the liver slices were washed and incubated with anti-IL-1β antibody (1:100 dilutions, #12703, CST, United States) for another 24 h. After wash three times, the slices were incubated with secondary antibody (1:100 dilutions) for 1 h the Nuclei of liver tissue were stained with DAPI. The immunofluorescence results were analyzed under fluorescence microscope (Olympus, Japan).

### Western blotting

Total proteins of liver samples were homogenized in RIPA (P0013C, Beyotime, China) lysis with Phenylmethylsulfonyl fluoride (PMSF, ST505, Beyotime, China) and phosphatase inhibitors. Protein concentrations were detected by BCA protein assay kit (P0012S, Beyotime, China). 30 μg protein samples were subjected to 12% SDS-PAGE (sodium dodecyl sulfate polyacrylamide gel electrophoresis). Then, the protein was transferred onto polyvinylidene fluoride (PVDF) membrane (IPVH00010, Millipore, United States). The membranes were blocking with 5% milk for 1 h. Subsequently, membranes were incubated overnight at 4°C with the specific primary antibodies (1:1000 dilutions) respectively. After washing three times, membranes were incubated with HRP-conjugated secondary antibody (1:100 dilutions) for 1 h. After washing, membranes were visualized with ECL chemiluminescent kit (BL523A, Biosharp, China). The blot values were calculated using ChemiDocTM MP Imaging system (Bio-Rad, United States).

### TUNEL staining

The numbers of the apoptosis cells in liver tissues was analyzed by TUNEL staining (11684817910, Roche, United States). Briefly, the samples were incubated with proteinase K (United States) for 15 min at 37°C. After washing, the slices were placed in a TUNEL solution mixture for 1 h. The apoptotic cell of liver sections was stained green. The results were observed under fluorescence microscope (Olympus, Japan).

### Statistical analysis

Data were represented as mean ± standard deviation. The normality of the data distribution has been checked by SPSS 12.0. The statistical differences of quantitative data were analyzed using ANOVA analysis followed by a post-test. The statistical differences of dichotomous data were analyzed using chi-squared test. The statistical process was performed with SPSS 12.0 software. Results were considered statistically distinct if *p* value <0.05.

## Results

### 3-TYP exacerbated the abnormity of liver histopathology and function in ALF mice

**FIGURE 1:**
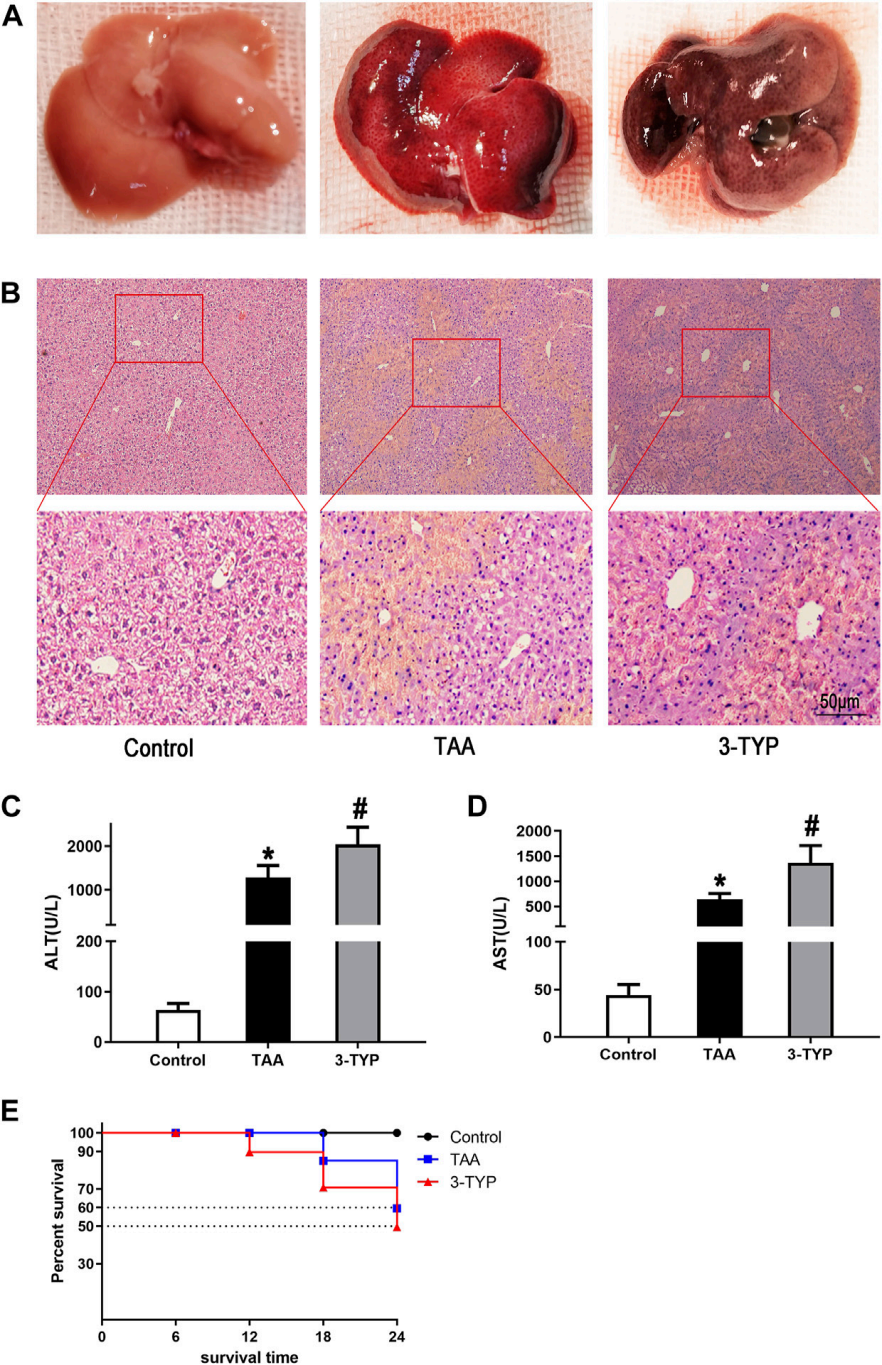
Effect of 3-TYP on liver damage and survival rate in each group, *n* = 10 per group. **(A)** The gross morphology of liver tissues. **(B)** HE staining of liver tissues. **(C)** serum ALT levels. **(D)** serum AST levels. **(E)** The 24 h survival rates of mice. *p < 0.05, *vs*. the control group. #*p* < 0.05, *vs*. the TAA group.

The gross morphology of livers in mice was performed in each group (Figure 1A). HE staining was used to measure the liver histopathology. As shown in the Figure 1B, in control group, the liver architecture is regular. Hepatocytes regularly arranged in hepatic lobule without necrosis and inflammatory cells. In TAA group, hepatic lobule structure was ruined and accompanied with massive necrosis and infiltrative inflammatory cells. However, in 3-TYP group, the necrosis area of liver tissue and inflammatory cells were further increased. In addition, the levels of liver enzyme of each group were assessed. Compared with control group, the levels of ALT and AST were significantly increased in TAA group and 3-TYP group, whereas the levels in 3-TYP group were higher than that in TAA group (Figures 1C,D). Finally, we observed the 24 h survival rate of all mice. The result showed that survival rate of TAA group at 24 h was 60.0%, whereas 3-TYP group was 50.0% (Figure 1E). These data indicated that 3-TYP exacerbated the liver damage in ALF mice.

### 3-TYP enhanced the oxidative stress markers and inflammatory reaction in ALF mice

**FIGURE 2:**
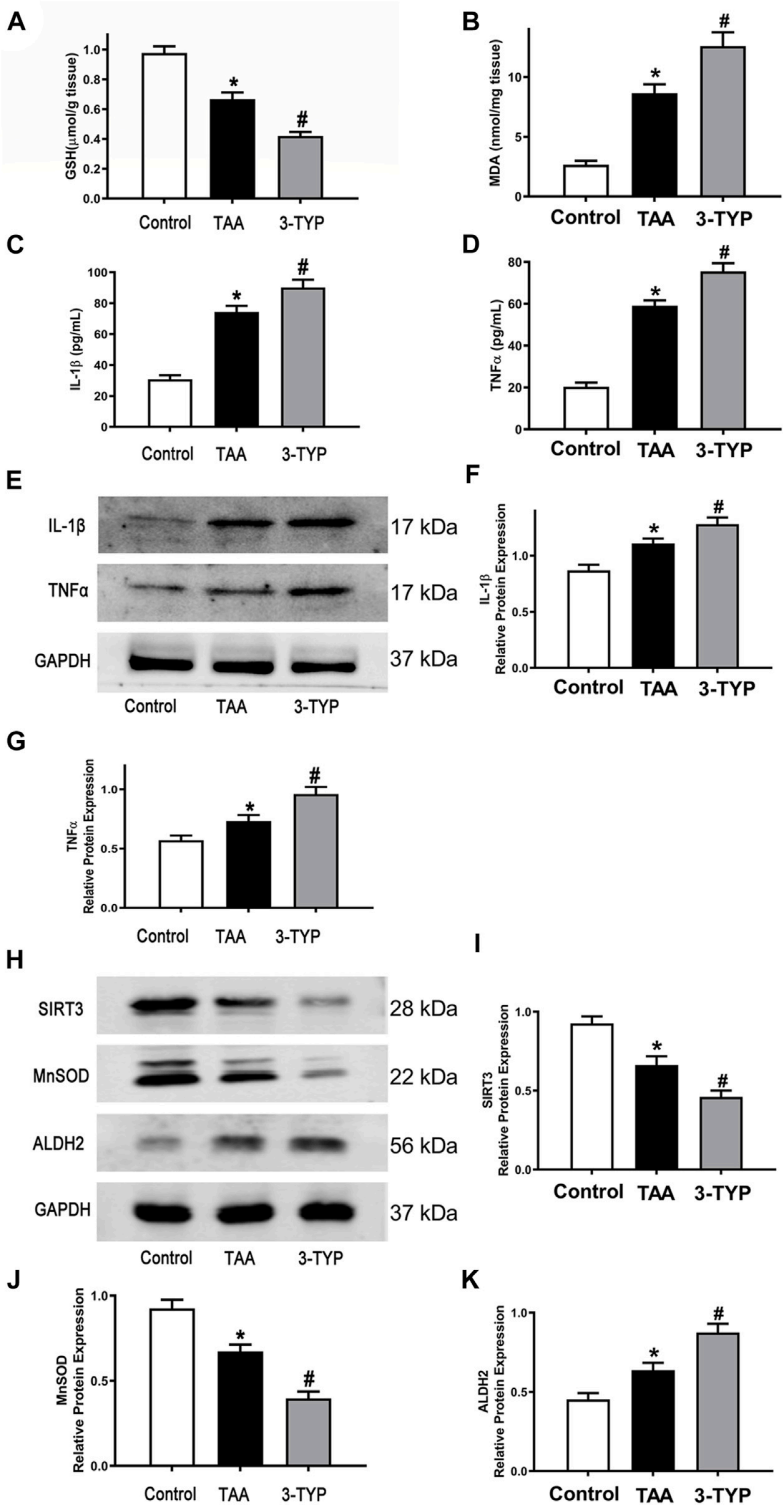
Effect of 3-TYP on inflammatory response in each group, *n* = 10 per group. **(A)** liver contents of GSH in liver tissues. **(B)** the MDA level in liver tissues. **(C)** the serum IL-1β levels. **(D)** the serum TNF-α levels. **(E)** The protein expressions of IL-1β and TNF-α in liver tissues were analyzed by western blotting. **(F)** The quantitative values of IL-1β. **(G)** The quantitative values of TNF-α. **(H)** The protein expressions of SIRT3, MnSOD, and ALDH2 in liver tissues were analyzed by western blotting. **(I)** The quantitative values of SIRT3. **(J)** The quantitative values of MnSOD. **(K)** The quantitative values of ALDH2. *p < 0.05, *vs*. the control group. #*p* < 0.05, *vs*. the TAA group.

**FIGURE 3:**
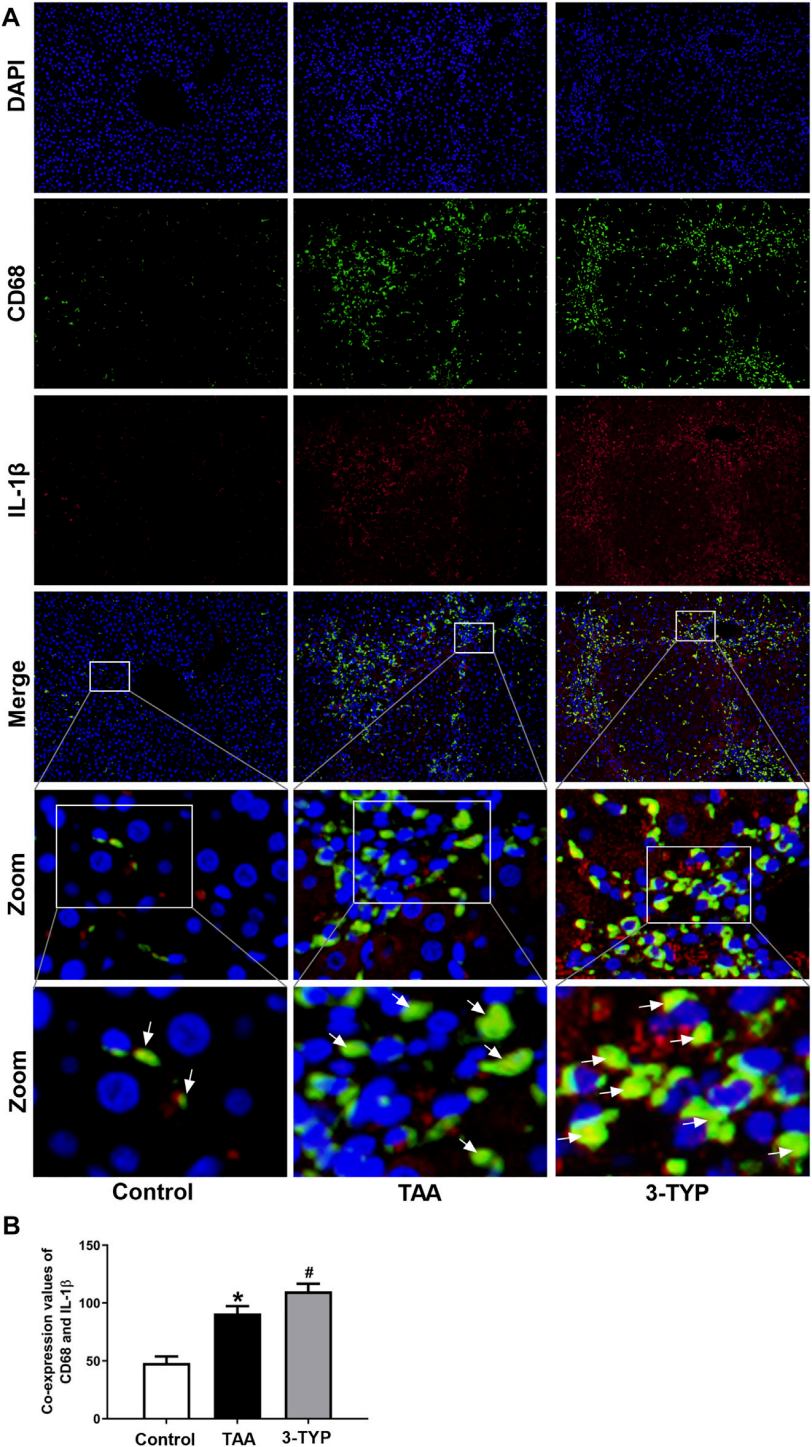
Effect of 3-TYP on the macrophage expression of IL-1β in each group, *n* = 10 per group. **(A)** Double immunofluorescent staining with macrophage marker anti-CD68 (green) and anti-IL-1β (red). **(B)** the quantitative co-expressions of CD68 and IL-1β. *p < 0.05, *vs*. the control group. #*p* < 0.05, *vs*. the TAA group. → the co-localization of CD68 and IL-1β.

The liver oxidative stress markers activities were determined. The liver contents of GSH were markedly decreased in TAA group, and the level was further decreased in 3-TYP group (Figure 2A). TAA administration resulted in remarkably increase in liver contents of MDA, compared to control group. While, 3-TYP further increased the contents of MDA (Figure 2B). Then, we measured the blood and liver samples of inflammatory cytokines. The results showed that the serum IL-1β and TNF-α levels were further elevated in 3-TYP, compared to TAA group (Figures 2C,D). Similarly, the proteins levels of IL-1β and TNF-α were significantly increased in 3-TYP group, compared to TAA group (Figures 2E–G). Then, the expression of SIRT3 and SIRT3 targets proteins (MnSOD, ALDH2) was determined by western blotting. The results showed that 3-TYP could significantly inhibit the expression of SIRT3, and regulate SIRT3 targets (Figures 2H–K). In addition, the cell type expression of IL-1β in liver tissue was detected by using double immunofluorescent staining. The primary antibody anti- IL-1β (red) and the macrophage marker anti-CD68 (green) were performed in this staining. Consistent with the western blotting results of IL-1β, the co-expressions of CD68 and IL-1β were significantly elevated in 3-TYP group in comparison with TAA group (Figures 3A,B). These results showed that 3-TYP enhanced the oxidative stress damage and inflammatory reaction in ALF mice.

### 3-TYP enhanced the MAPK and NF-KB pathways in ALF mice

**FIGURE 4:**
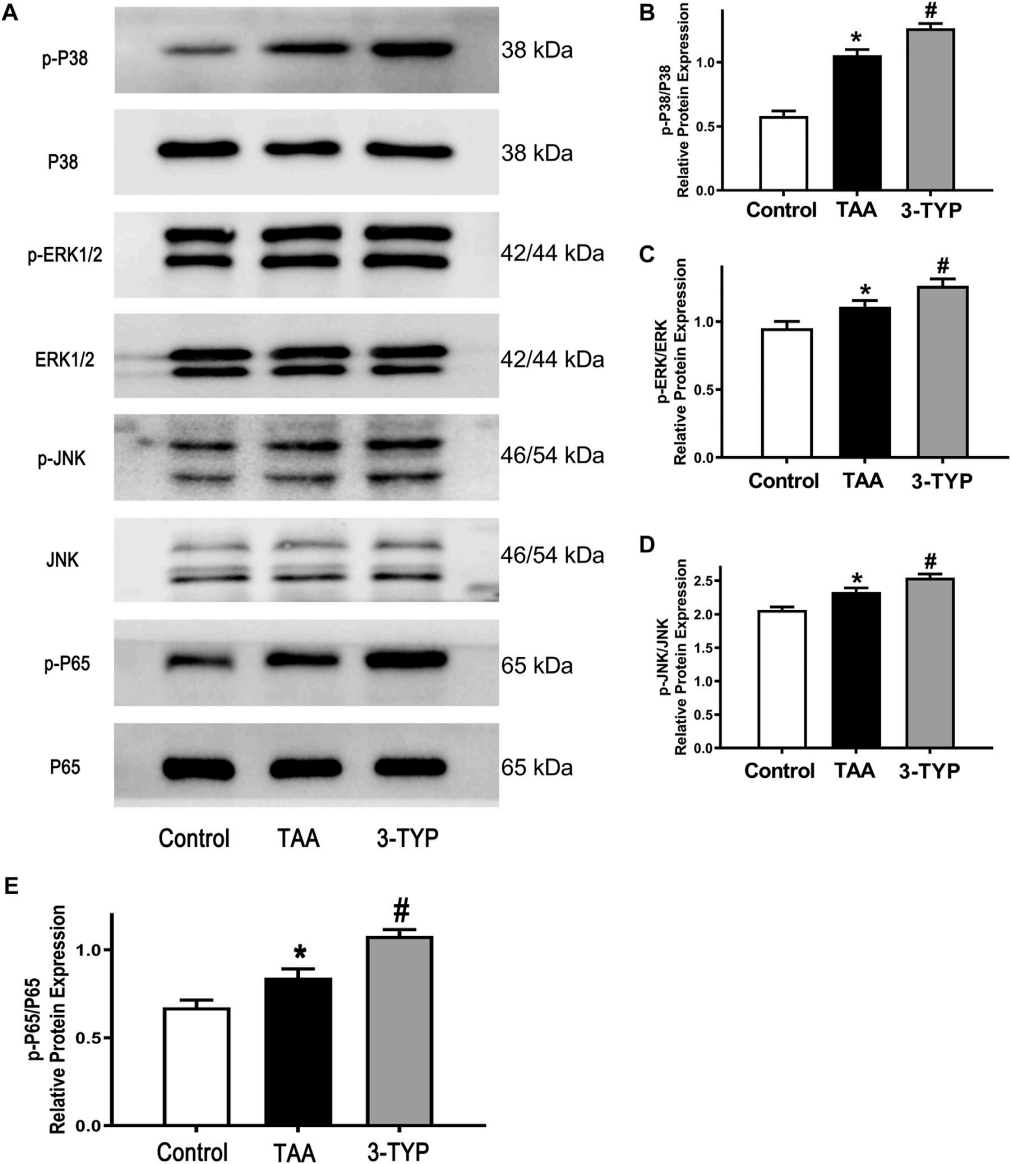
Effect of 3-TYP on activation of MAPK and NF-κB pathways in each group, *n* = 10 per group. **(A)** The expressions of p-P38, P38, p-ERK1/2, ERK1/2, p-JNK, and JNK in liver tissues were detected by western blotting. **(B)** The quantitative values of p-P38/P38. **(C)** The quantitative values of p-ERK/ERK. **(D)** The quantitative blots of p-JNK/JNK. **(E)** The quantitative blots of p-P65/P65. *p < 0.05, *vs*. the control group. #*p* < 0.05, *vs*. the TAA group.

The MAPK and NF-κB pathways play an important role in regulating inflammatory process. In order to the impact of 3-TYP on MAPK and NF-κB pathway, we examined the relative proteins expression on liver tissue. The results showed that the expressions of p-P38, p-ERK1/2, and p-JNK were significantly elevated in TAA group, compared with control group. However, 3-TYP treatment increased these proteins expressions, compared with TAA group (Figures 4A–D). In addition, we detected the changes of NF-κB pathways. As expected, 3-TYP accelerated the expression of phosphorylation of NF-κB p65 in 3-TYP group, compared to TAA group (Figure 4E). The data suggested that 3-TYP promoted the activation of MAPK and NF-κB pathways in ALF mice.

### 3-TYP enhanced oxidative stress and endoplasmic reticulum stress in ALF mice

**FIGURE 5:**
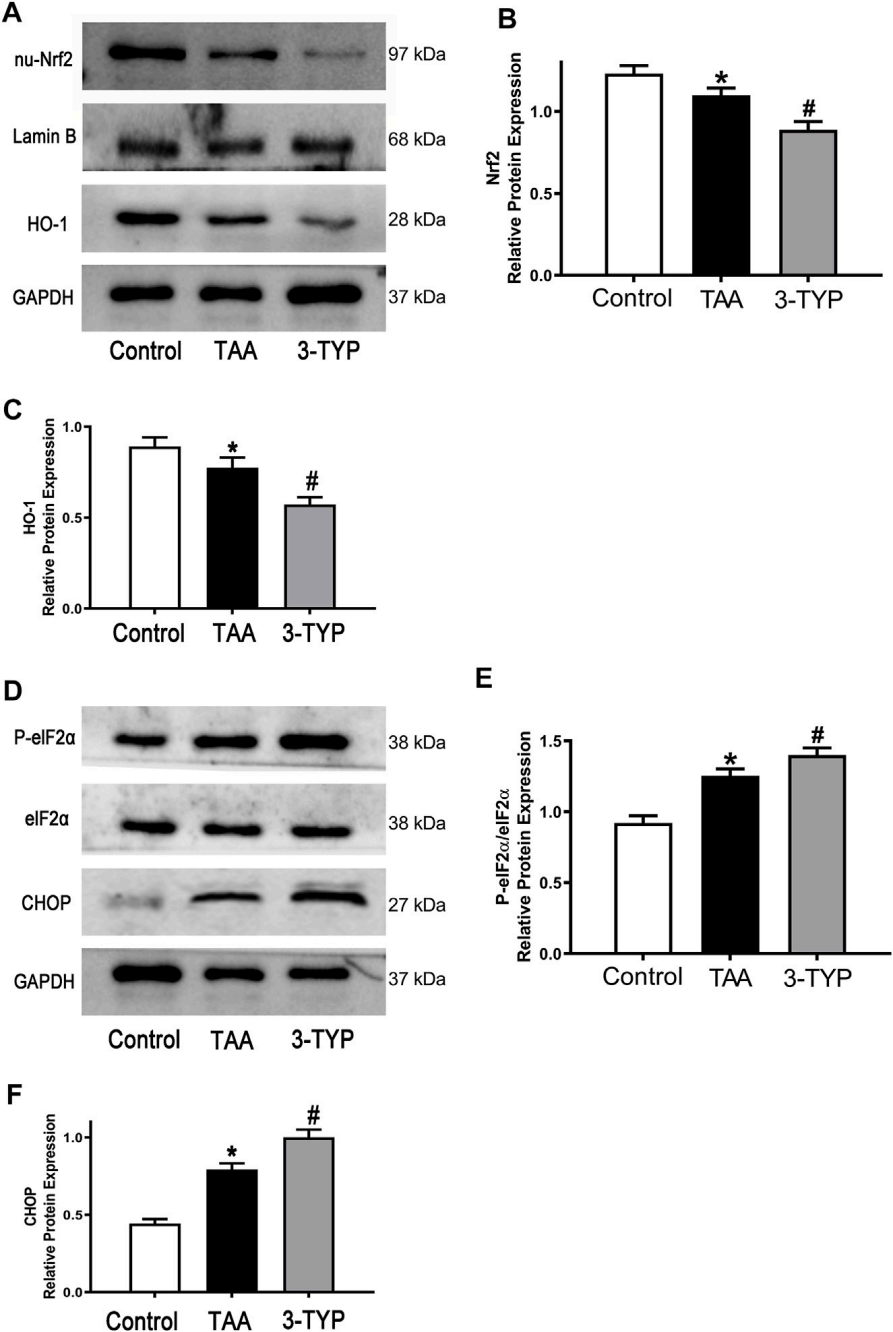
Effect of 3-TYP on activation of oxidative stress and endoplasmic reticulum stress in each group, *n* = 10 per group. **(A)** The expressions of Nrf2 and HO-1 in liver tissues were analyzed by western blotting. **(B)** The quantitative blots of Nrf2. **(C)** The quantitative blots of HO-1. **(D)** The expressions of p-elF2α, elF2α, and CHOP in liver tissues were measured by western blotting. **(E)** The quantitative values of p-elF2α/elF2α. **(F)** The quantitative values of CHOP. *p < 0.05, *vs*. the control group. #*p* < 0.05, *vs*. the TAA group.

Oxidative stress and endoplasmic reticulum stress were considered as the important mechanism of liver injury. The changes of oxidative stress makers (MDA and GSH) were observed in above text. We next examined the expressions of oxidative stress relative proteins (Nrf2/HO-1). We found that antioxidant factors Nrf2 and HO-1 were significantly reduced in TAA group, while the proteins were further decreased in 3-TYP group (Figures 5A–C). Similarly, we explored the expressions of endoplasmic reticulum stress relative proteins. The expressions of p-elF2α, elF2α, and CHOP in TAA group were significantly increased, compared with control group. However, 3-TYP administration further promoted these proteins expressions (Figures 5D–F). The results indicated that 3-TYP enhanced the oxidative stress and endoplasmic reticulum stress in ALF mice.

### 3-TYP increased the hepatocyte apoptosis in ALF mice

**FIGURE 6:**
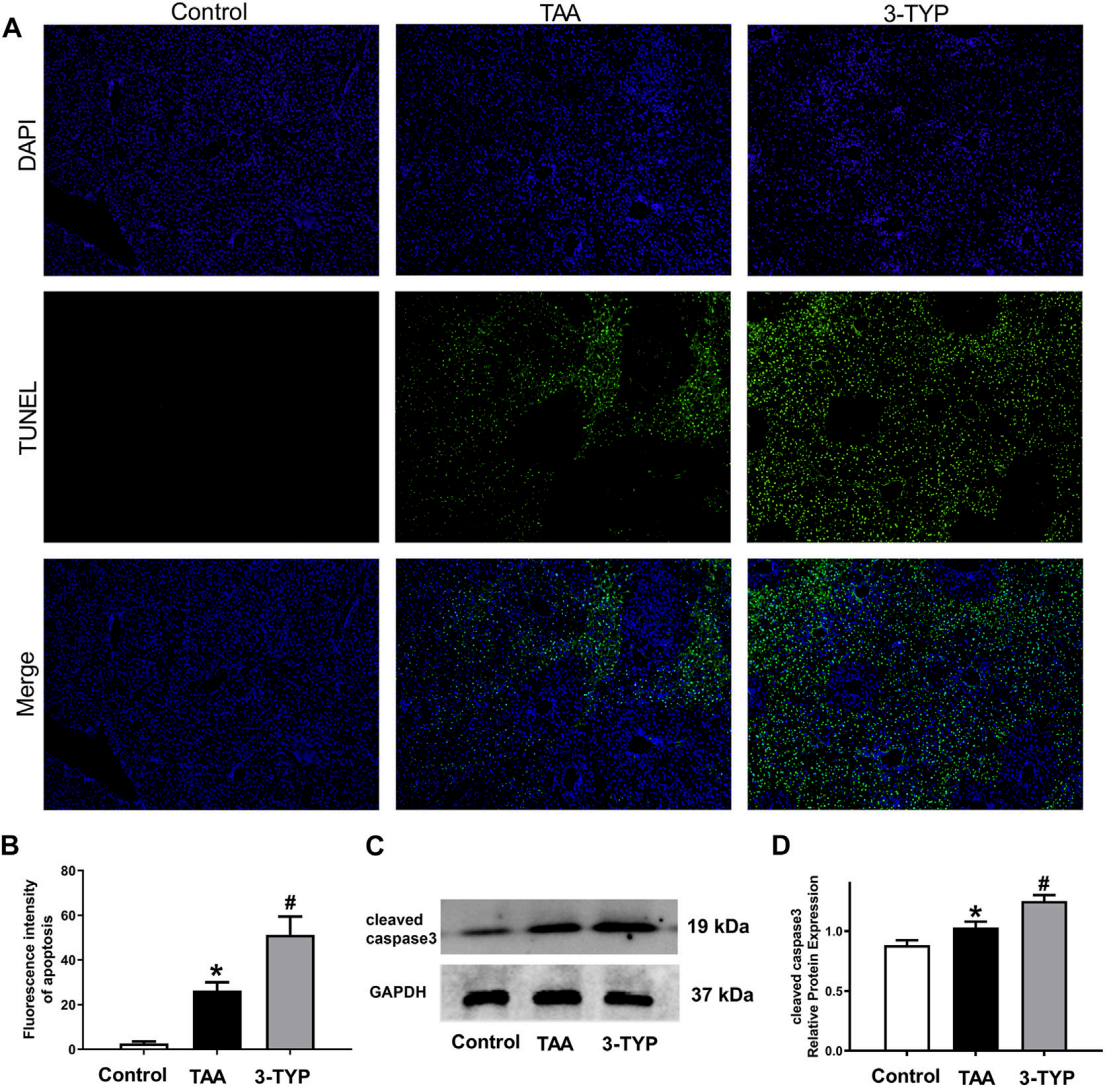
Effects of 3-TYP on liver cell apoptosis in each group, *n* = 10 per group. **(A)** The apoptosis cells of liver samples were measured by TUNEL staining. **(B)** Quantification of apoptosis. **(C)** The expressions of cleaved caspase 3 were detected by western blotting. **(D)** The quantitative values of cleaved caspase 3. *p < 0.05, *vs*. the control group. #*p* < 0.05, *vs*. the TAA group.

As mentioned above, massive necrosis was observed in TAA group liver by HE staining. In order to confirm the number of necrotic cells, TUNEL staining was performed. The staining results confirmed that the numbers of apoptosis cells were obviously elevated in ALF mice. However, the numbers of apoptosis cells were further increased in 3-TYP group (Figures 6A,B). Then, we examined the expressions of cleaved caspase 3 in liver tissues. The results showed that cleaved caspase 3 was significantly increased in TAA group, compared with control group. Treatment with 3-TYP further increased the expression (Figures 6C,D). These results showed that 3-TYP increased the hepatocyte apoptosis in ALF mice.

## Discussion

The aim of this study is to investigate the effects of selective SIRT3 inhibitor 3-TYP on thioacetamide-induced ALF mice and explore the possible mechanisms. SIRT3 is the first recognized sirtuin which is localized to mitochondria (Michishita et al., 2005). SIRT3 is expressed in various tissues, including heart, brain, kidneys, liver and testes (Jin et al., 2009). With the deep-going research of SIRT3, the multiple physiological functions are recognized. Some studies reported that SIRT3 was closely linked with inflammation. A study showed that SIRT3-defcient mice increased the inflammation response and NLRP3 inflammasome activation in endotoxin-induced acute lung injury (Kurundkar et al., 2019). Another study reported that SIRT3 knockout showed inflammation in the heart of mice, and SIRT3 overexpression inhibited the inflammatory response in TNF-α-induced human cardiac cells (Palomer et al., 2020). Similarly, SIRT3 decreased palmitate-induced inflammation in proximal tubular cells (Koyama et al., 2011). In previous study, 3-TYP, a SIRT3-selective inhibitor, has been reported to aggravate oxidative damage and reduce enzymatic activity of MnSOD2 in the hair cells of mice (Liang et al., 2021). 3-TYP can not only eliminate the cardioprotective effects of melatonin (Zhai et al., 2017), but also activate NF-κB and NLRP3 pathways, reducing survival rate in mice (Lv et al., 2021). These results are consistent with our study. Our results showed that 3-TYP exacerbated the abnormity of liver histopathology and function in ALF mice. 3-TYP could effectively downregulate the expression of SIRT3, resulting in the increase of pro-inflammatory cytokines TNF-α and IL-1β in ALF mice. Consistent with this, a study about CCl4-induced liver injury reported that SIRT3 KO mice showed more serious damage of histopathology and function than wild-type (WT) mice (Li et al., 2019). The adverse effects of 3-TYP on cells or animals have been widely reported. However, very few studies have shown that 3-TYP can reduce some inflammatory molecules under the influence of some drugs (Sun et al., 2022). This may be due to the effects of the combined drugs, or the effect of the dose and duration of 3-TYP. Concentration and time gradient and other SIR3 inhibitors are not used in present study. This is a limitation of our results.

It is well-known that MAPK and NF-κB signaling pathways regulate the inflammatory process in various inflammatory diseases (Arthur and Ley, 2013). Numerous evidences indicated that the activation of MAPK pathways involved in multiple liver injury models (Sun et al., 2017; Zhang et al., 2017). A variety of drugs can reduce liver inflammation and damage by acting on MAPK and NF-κB, and have a protective effect on liver (Gao et al., 2020; Cai et al., 2021). In addition, a study showed that overexpression of SIRT3 repressed palmitate-induced MAPK signaling activation in pancreatic *β*-cell (Kim et al., 2015). Another study reported that upregulation of SIRT3 inhibited the phosphorylation of p38 in ischemia-reperfusion (I/R)-induced endothelial cell (Zhao et al., 2019). Therefore, SIRT3 is closely related to MAPK signaling pathway. In order to determine whether SIRT3 inhibitor 3-TYP can affect MAPK signaling, we detected the phosphorylation of three MAPKs. The results showed that inhibition of SIRT3 by 3-TYP could enhance the phosphorylation of three MAPKs. In addition, 3-TYP increased the levels of transcription factor phosphorylation of NF-κB p65. MAPK and NF-κB signaling pathways have important effects on inflammation and cell death. Increased levels of NF-κB p65, MAPK (p38, ERK1/2 and JNK) phosphorylation were reported in septic acute kidney injury mice (Ren et al., 2020). Besides, extracellular tumor necrosis factor and reactive oxygen species could induce apoptosis by activating p38 MAPK kinase (Anne et al., 2013). Based on these results, we propose that 3-TYP might aggravate the inflammation in TAA-treated mice by upregulating MAPK and NF-κB pathways.

Oxidative stress reaction can produce products such as ROS and activate a variety of transcription factors, leading to differential expression of some genes in inflammatory pathways, resulting in tissue damage, mainly manifested in inflammation and oxidative damage of DNA, proteins and lipids by ROS and other oxides (Hussain et al., 2016). The antioxidant effect of SIRT3 has been widely studied in a variety of diseases. A previous study demonstrated that Honokiol (an agonist of SIRT3) improved the oxidative stress injury in hyperglycemic rats after intracerebral hemorrhage (ICH) (Zheng et al., 2018). Overexpression of SIRT3 inhibited H2O2-induced oxidative stress damage *via* regulating SOD2 levels and activity (Ma et al., 2020). Similarly, upregulation of SIRT3 decreased tert-butyl hydroperoxide (t-BHP)-induced oxidative injury in AML12 hepatocytes (Liu et al., 2017). Consistent with those studies, our results found that the oxidative stress markers MDA was obviously elevated after 3-TYP treatment. The levels of GSH were significantly decreased in this process. Moreover, the decrease of anti-oxidant factors Nrf2 and HO-1 were observed in TAA-treated mice after 3-TYP administration, indicating that 3-TYP might aggravate liver injury through increasing oxidative stress. Inflammation caused by oxidative stress is the cause of many diseases. The deficiency of antioxidants such as vitamin C, vitamin E and GSH or the decrease of antioxidant enzymes will directly affect the antioxidant capacity of the body. Activation of Nrf2/HO-1 signaling is also an important mechanism against oxidative stress (Zhang et al., 2021).

As one of the mechanisms of liver injury, ERS has also been extensively studied (Dara et al., 2011). Some studies have reported that SIRT3 is involved in ERS. A study found that SIRT3 overexpression inhibited the palmitate-induced ERS in pancreatic *β*-cells, and SIRT3 siRNA promoted the cells injury (Zhang et al., 2016). Knockdown of SIRT3 partially prevented the protective effect of resveratrol on ERS in HT22 cells (Yan W. J. et al., 2018). In order to verify whether 3-TYP can affect ERS, we detected eIF2α/CHOP pathway, which is an important pathway of ERS. The results showed that inhibition of SIRT3 resulted in the increase of ERS-related protein levels in liver injury mice, suggesting that 3-TYP may partially play a role on ERS. ERS is a protective response that restores protein homeostasis through activation of the unfolded protein response (UPR). However, UPR can lead to cell death under severe ERS. ERS can activate NLRP3 inflammasome and induces inflammatory responses through oxidative stress, calcium homeostasis, and NF-κB activation. ERS-induced inflammasome activation is the pathological basis of various inflammatory diseases (Li et al., 2020). Therefore, ERS is an important factor in promoting inflammation induced by 3-TYP. Next, we evaluated the effect of 3-TYP on hepatocyte apoptosis in TAA-treated mice. As expected, 3-TYP promoted the apoptosis of liver cells in ALF mice. Similarly, previous experiments have reported that Sirt3-transgenic (TG) mice alleviated high-fat-induced hepatocyte apoptosis (Li et al., 2018). However, in the study of HCC cells lines, upregulation of SIRT3 using adenovirus promoted the apoptosis of HepG2 and HuH-7 cells (Zhang and Zhou, 2012). The opposite effects of SIRT3 on apoptosis may due to the different characteristics of primary hepatocytes and tumor cells.

In summary, SIRT3 inhibitor 3-TYP exacerbates liver histopathological and functional damages, increase the inflammatory responses and aggravate the hepatocyte apoptosis in ALF mice. This work also has some limitations. The age of mice in this study is equivalent of teenagers, not young adults (the most common age for acute liver failure). Another limitation is that concentration and time gradient and other SIR3 inhibitors are not designed. For these reasons, further studies need to be explored.

## Data Availability

The original contributions presented in the study are included in the article/Supplementary material, further inquiries can be directed to the corresponding author.
